# The Splicing Factor SR45 Negatively Regulates Anthocyanin Accumulation under High-Light Stress in *Arabidopsis thaliana*

**DOI:** 10.3390/life13061386

**Published:** 2023-06-14

**Authors:** Mohammed Albaqami

**Affiliations:** Department of Botany and Microbiology, College of Science, King Saud University, P.O. Box 2455, Riyadh 11451, Saudi Arabia; mmbaqami@ksu.edu.sa

**Keywords:** environmental stress, light stress, photoinhibition, anthocyanin, RNA splicing, SR proteins, SR45, *Arabidopsis*

## Abstract

High-intensity light (HL) greatly induces the accumulation of anthocyanin, a fundamental compound in photoprotection and antioxidation. Many mechanisms regulating anthocyanin biosynthesis are well-characterized across developmental and environmental conditions; however, post-transcriptional regulation of its biosynthesis remains unclear. RNA splicing is one mechanism of post-transcriptional control and reprogramming in response to different developmental cues and stress conditions. The *Arabidopsis* splicing modulator SR45 regulates a number of developmental and environmental stress responses. Here, we investigated the role of SR45 and its isoforms in HL-induced anthocyanin accumulation. We found that the *SR45* promoter contains light-responsive *cis*-elements, and that light stress significantly increases *SR45* expression. Furthermore, we found that mutant plants lacking SR45 function (*sr45*) accumulate significantly more anthocyanin under HL. *SR45* is alternatively spliced to produce two proteins, SR45.1 and SR45.2, which differ by seven amino acids. Intriguingly, these isoforms exhibited distinct functions, with only SR45.1 reversing anthocyanin accumulation in the *sr45* plants. We also identified possible SR45 target genes that are involved in anthocyanin synthesis. Consistent with the antioxidant role of anthocyanin, we found that *sr45* mutants and *SR45.2* overexpression lines accumulate anthocyanin and better tolerate paraquat which induces oxidative stress. Collectively, our results reveal that the *Arabidopsis* splicing regulator SR45 inhibits anthocyanin accumulation under HL, which may negatively affect oxidative stress tolerance. This study illuminates splicing-level regulation of anthocyanin production in response to light stress and offers a possible target for genetic modification to increase plant stress tolerance.

## 1. Introduction

One of the environmental conditions that restricts plant growth and development is excessive light stress, which occurs when a plant receives light beyond its photosynthetic capability. Overexcitation of the photosystem machinery causes a phenomenon known as “photoinhibition”, which alters the redox state of the photosystem and increases reactive oxygen species (ROS) production [1,2]. High ROS accumulation results in oxidative damage to proteins and lipids in the photosystem apparatus which, in turn, leads to severe cellular damage and retardation of plant growth [3,4]. Plants use both enzymatic and non-enzymatic ROS avoidance mechanisms to maintain low ROS levels and proper growth and development [5,6]. The enzymatic antioxidant system is comprised of superoxide dismutase (SOD), catalase (CAT), ascorbate peroxidase (APX), and glutathione peroxidase (GPX), while the non-enzymatic antioxidant system consists of ascorbate, glutathione, carotenoids, tocopherols, and phenolic compounds (including flavonoids and anthocyanins).

Anthocyanin accumulation is the normal response to severe light stress. Anthocyanins have antioxidant characteristics in addition to protecting the photosystem from light damage by absorbing excessive light, thereby limiting the energy input to the photosystem machinery [7,8,9,10,11]. Many abiotic stimuli, including salt, osmotic, and drought stress, as well as increased temperatures and light, induce anthocyanin accumulation in a tissue-dependent manner [12,13,14]. Notably, mutants lacking in anthocyanin exhibit abnormal development patterns under both normal and stressful conditions [15,16]. Conversely, anthocyanin over-accumulation mutants have greater resistance to stressors such as HL and drought [17,18].

Thorough investigation of the molecular underpinnings of the anthocyanin biosynthesis pathway has revealed many transcription factor (TF) families as transcriptional regulators of anthocyanin biosynthesis [19,20,21,22]. In particular, members of the MYB, basic helix–loop–helix (bHLH), and WD40 repeat protein families form the MBW complex, which positively regulates expression of the genes responsible for anthocyanin production in a light-dependent manner [23,24]. In *Arabidopsis*, the MBW complex is formed through the interaction of a bHLH TF (TRANSPARENT TESTA 8 [TT8], GLABRA 3 [Gl3], or ENHANCER OF GLABRA 3 [EGL3]), a WD40 repeat protein (TRANSPARENT TESTA GLABRA1 [TTG1]), and MYB transcription factors (MYB75, MYB90, MYB113, or MYB114) [25,26], which promote the expression of genes late in the anthocyanin synthesis pathway [27].

Anthocyanin production in response to environmental cues is further mediated by several other regulatory genes. In *Arabidopsis*, for instance, the accumulation of anthocyanin is reduced by a high level of MYBL2, which is negatively regulated by HL, thereby allowing light-induced anthocyanin accumulation [28]. At the post-transcriptional level, miR156c targeting of the *SQUAMOSA PROMOTER BINDING PROTEIN-LIKE9* (*SPL9*) gene negatively regulates anthocyanin accumulation by destabilizing the MBW complex and downregulating anthocyanin biosynthesis genes [29].

Alternative splicing (AS) is a post-transcriptional mechanism that regulates gene expression and controls most eukaryotic biological processes. Genome-wide studies have shown that regulation of AS helps plants adapt to a range of environmental conditions, including abiotic stresses [30,31,32,33,34]. Indeed, in both vascular and non-vascular plants, light modifies AS all over the genome to fine-tune gene expression and metabolic processes in a light-responsive manner [35,36,37]. In *Arabidopsis*, the photoreceptors phytochrome A (phyA) and phytochrome B (phyB) regulate the splicing and expression of around 1500 genes in response to red and far-red light [38]. However, light-responsive RNA splicing seems to also be controlled through a retrograde signal pathway that is dependent on a molecule intermediately synthesized in the chloroplast during photosynthesis [39,40]. Additionally, when *Arabidopsis* plants grown under a 12 h light/dark cycle were given a short pulse of white or red light during the dark period, this resulted in AS modulation of about 380 genes [36]. Most of those genes are involved in cellular processes such as basic metabolism, stress response, circadian clock regulation, and RNA processing/splicing. This provides evidence that AS, which is influenced by light, contributes to photomorphogenesis in plants.

It has also been established that AS regulates the metabolism of phenylpropanoids and their downstream metabolites, such as flavonoids, which include anthocyanin. In particular, it has been shown in kiwifruit (*Actinidia chinensis*) that AS regulates numerous anthocyanin biosynthesis genes during fruit growth and ripening, including *flavanone 3-hydroxylase* (*F3H*), *chalcone synthase* (*CHS*), *dihydroflavonol-4-reductase* (*DFR*), *anthocyanidin synthase* (*ANS*), and *uridine diphosphate* (*UDP*)-*glucosyltransferase* (*UGT*) [41]. In tea plants, it has further been found that about 90 genes in the anthocyanin biosynthesis pathway are alternatively spliced and the expression of a certain isoform of these factors is important for the accumulation of anthocyanin [42].

In broad terms, AS may offer a regulatory layer to either stimulate or suppress anthocyanin production by creating shortened transcripts with varying degrees of functionality. For instance, AS of the gene encoding the MYB TF BnaPAP2, which regulates anthocyanin biosynthesis in rapeseed (*Brassica napus* L.), results in distinct transcripts with opposing functions [43]. The truncated isoforms were found to be unable to interact with the bHLH protein *in vitro* and to downregulate the expression of anthocyanin biosynthesis regulatory genes when overexpressed in *Arabidopsis* [43]. It has also been shown that AS regulation of anthocyanin biosynthetic genes is responsive to both biotic and abiotic stressors. For example, iron deficiency in *Arabidopsis* alters the splicing patterns of genes implicated in anthocyanin production, such as those encoding phenylalanine ammonia lyase (PAL) and coumarate CoA ligase (4CL) [44].

Serine and arginine-rich (SR) proteins, which comprise a family of RNA-binding proteins, are involved in many different aspects of RNA metabolism, most notably RNA splicing. One *Arabidopsis* SR that has been thoroughly studied for its role in the regulation of plant growth and environmental stimulus responses is SR45 [45,46,47,48]. Loss of function of SR45 causes a variety of developmental defects, including abnormal flowering time, flower phenotype, and inhibition of root growth [45,49]. Moreover, mutant (*sr45*) plants exhibit a variety of biotic and abiotic stress response phenotypes, including hypersensitivity to high salinity and sugar [46,47,50]. It is also worth noting that *SR45* is alternatively spliced to produce two isoforms, *SR45.1* and *SR45.2*, with SR45.1 having seven additional amino acids compared to SR45.2 [51]. Interestingly, genetic complementation of *sr45* with the individual isoforms has revealed them to have distinct functions, whether in plant development or stress response [51,52]. In addition, genome-wide identification of SR45 targets has shown that SR45.1 binds directly or indirectly to thousands of *Arabidopsis* transcripts that function in numerous biological processes [53].

In this study, we explore the functions of *Arabidopsis* SR45 in HL stress response and how this splicing factor influences the preeminent response, anthocyanin accumulation. Our research revealed that the *SR45* promoter contains several light- and stress-responsive elements and *SR45* expression is strongly up-regulated by light intensity. Loss of SR45 function results in increased anthocyanin accumulation under HL conditions, while lines over-expressing individual *SR45* isoforms (*SR45.1* and *SR45.2*) showed distinct light stress response phenotypes, with the *SR45.1* line accumulating less anthocyanin under light stress than the *SR45.2* line. We additionally demonstrated that SR45 binds to and controls the expression of several gene transcripts involved in light-induced anthocyanin production. Furthermore, we aimed to investigate the response of all genotypes to paraquat (PQ), a well-known herbicide that induces ROS, causes oxidative damage, and enhances anthocyanin accumulation in plants [54]. We found that seedlings of the mutant and the *SR45.2* over-expression line each accumulate high levels of anthocyanin in response to PQ, and both lines are more resistant to PQ than the WT and *SR45.1* over-expression plants. Overall, our results suggest that SR45 is critical for light responses in plants, with potential implications in the control of anthocyanin production and ROS defense systems.

## 2. Materials and Methods

### 2.1. Plant Materials and Growth Conditions

In this study, the ecotype Columbia (Col-0) of *Arabidopsis thaliana* was used as the wild type (WT). The *sr45* mutant that was used in this study—and exhibited loss of function—was described in [45]. The lines over-expressing *SR45.1* and *SR45.2* in the mutant background were described in [51]. All lines were grown on soil in a controlled environment at a temperature of 22 °C with a light intensity of 120 μmol m^−2^ s^−1^ and a 12:12 light/dark cycle. All seeds used in this study originated from the same planting batch to ensure uniformity. Seeds were always cold treated at 4 °C for 3 days, germinated on 0.5× Murashige and Skoog (MS) medium for 7 days in a controlled growth room with 22 °C, 120 mol/m^2^ of light intensity, and a 12:12 light/dark cycle, and then finally, planted in soil.

### 2.2. Promoter Analysis

We obtained the DNA sequence 1 kb upstream of *SR45* from the Arabidopsis Information Resource (TAIR) “https://www.arabidopsis.org/ (accessed on 19 June 2022)”, and then examined regulatory *cis*-elements in the *SR45* promoter using PlantCARE “http://bioinformatics.psb.ugent.be/webtools/plantcare/html/ (accessed on 19 June 2022)”.

### 2.3. Light Stress Treatment

We used the high-light treatment protocol described in [55] with some modifications. All lines were grown on MS medium for 10 days before being transplanted into soil, and then grown for another 21 days at 22 °C in a controlled environment under 120 mol/m^2^ of light and a 12:12 light/dark cycle. After that, whole plants on soil from each line were placed for 3 days in a growth chamber with continuous high-intensity light (400 mol m^−2^ s^−1^), whereas control plants from each line were left in the lower light intensity of 120 μmol m^−2^ s^−1^ and a 12:12 light/dark cycle. At least 30 plants from each line were used in each of the 3 biological replicates.

### 2.4. Paraquat (PQ) Treatment

We followed the paraquat treatment procedure provided by [56]. The seeds of all lines were first subjected to sterilization and cold treatment as described above, and then, they were germinated on 0.5× MS medium either with or without 0.2 μM paraquat. Subsequently, the plates were placed in an incubator for 2 weeks at a temperature of 22 °C with a light intensity of 120 mol m^−2^ s^−1^ and a light/dark cycle of 12:12. Following that, the root length of the seedlings was measured, and photographs were taken.

### 2.5. RNA-seq Data and Gene Expression Analysis

The RNA-seq data utilized in this research was obtained from a previous work published in *Cell Reports* [57]. Briefly, 7-day-old *Arabidopsis* seedlings (Columbia, Col-0) were grown in MS media and subjected to continuous high light (HL, 1200 mmol m^−2^ s^−1^), with control plants kept under the growth light condition (GL, 60 mmol m^−2^ s^−1^). Samples were collected at time periods of 0.5 h, 6 h, 12 h, 24 h, 48 h, and 72 h, after which HL plants were returned to the GL condition and a recovery sample was collected after 14 h. Total RNA was extracted using the RNAeasy Plant Mini Kit (QIAGEN), and RNA-seq libraries were prepared using 4 mg of total RNA and the TruSeq Stranded mRNA Library Prep Kit (Illumina, San Diego, CA, USA). Subsequently, single-end RNA-seq was generated on an Illumina HiSeq 2500 sequencing machine at the Next-Generation Sequencing Core of the Salk Institute for Biological Studies. The raw counts of each gene were determined using the BRB Digital Gene Expression (BRB-DGE) program (https://arraytools.github.io/bdge/).

### 2.6. Total RNA Extraction and RT-PCR

Total RNA was extracted from 21-day-old wild type (WT), *sr45*, *sr45::SR45.1*, and *sr45::SR45.2* plants grown in HL or GL, as described above, using the RNeasy Plant Mini Kit (Qiagen, Hilden, Germany) according to the manufacturer’s instructions for quantitative real-time PCR (qPCR) analysis. Residual genomic DNA was eliminated from the samples using DNase I (Fermentas, Hanover, MD, USA) in accordance with the manufacturer’s instructions. SuperScript III (Invitrogen, Carlsbad, CA, USA) was used to synthesize cDNA in a 20 μL reaction per the manufacturer’s instructions, and qPCR was performed using a LightCycler 480 (Roche Applied Science, Penzberg, Germany) instrument with the LightCycler 480 SYBR Green 1 master mix. The PCR program conditions were as follows: 3 min at 95 °C, followed by 44 cycles of 10 s at 95 °C, 30 s at 60 °C, and 30 s at 72 °C. *ACTIN-2* was used as an internal control for gene expression analysis. The primers utilized in this work are detailed in Supplementary Table S1 and were generated using PRIMER3Plus “www.primer3plus.com (accessed on 1 September 2022)”.

### 2.7. Chlorophyll Content Measurement

For the purpose of measuring total chlorophyll, random leaves from all genotypes were collected and processed after 72 h of growth under GL or HL conditions according to the procedures described in [58]. Briefly, we collected and weighed leaf samples, then ground them into powder using a mortar and pestle chilled with liquid nitrogen. Chlorophyll was then extracted using 80% (*v*/*v*) acetone, and cell debris was removed by centrifuging at 10,000× *g* for 15 min at 4 °C. The chlorophyll concentration was determined using the spectrophotometric method detailed in [59].

### 2.8. Anthocyanin Content Measurement

Anthocyanin content was measured as described in [60]. In short, we collected and weighed random leaf samples or whole seedlings from all genotypes after 72 h of growth in GL or HL conditions, with or without 0.2 uM PQ, then ground them under liquid nitrogen and extracted the resulting powder with 99:1 methanol:HCl (*v*/*v*) at 4 °C overnight. The relative anthocyanin levels for each sample were calculated using the formula: OD_530_ − (0.25 OD_657_) × extraction volume (mL) × 1/weight of tissue sample (g).

### 2.9. Statistical Analysis

To determine statistical significance, we conducted statistical analyses using the Student’s *t*-test integrated into Microsoft Excel software, unless otherwise specified. A significance level of *p* < 0.05 was used to determine statistical significance. All experiments were performed at least three times to account for technical and biological variability. Mean values ± standard error (SE) were presented for all measurements, and a *p*-value of less than 0.05 was considered indicative of a significant difference.

### 2.10. Accession Numbers 

The sequence data from this article can be found at TAIR “https://www.arabidopsis.org (accessed on 19 June 2022)” under accession numbers: AT1G16610 (*SR45*), AT4G31877 (*MIR156C*), AT2G42200 (*SPL9*), AT5G08640 (*FLS1*), and AT3G18780 (*ACT2*).

## 3. Results

### 3.1. Expression of SR45 Is Triggered by High-Light Stress

**Figure 1:**
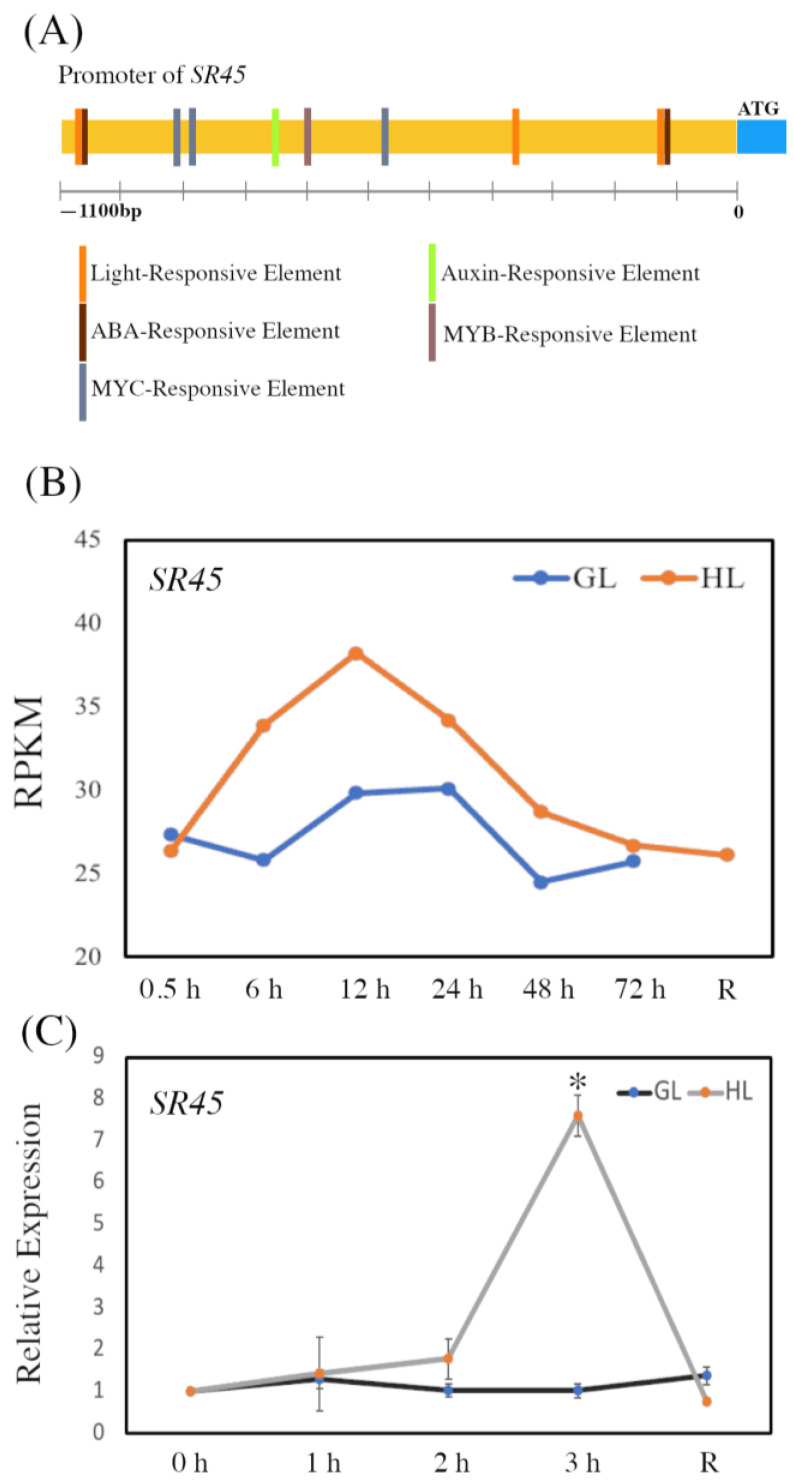
Expression of *SR45* in response to high light (HL). (**A**) *Cis*-regulatory elements identified in the *SR45* promoter. The 1100 bp DNA fragment upstream of the ATG start codon of *SR45* was examined with PlantCARE. Colors indicate different regulatory elements. (**B**) Expression of *SR45* under HL and growth light (GL) conditions based on RNA-seq data [57]. The *x*-axis indicates the time points and after recovery (R). The *y*-axis represents the average reads per kilobase per million mapped reads (RPKM). (**C**) qPCR analysis of *SR45* under HL. The *x*-axis indicates time points and after recovery (R). The *y*-axis represents relative expression. Asterisks indicate a significant difference (* *p* < 0.05) determined by a *t*-test.

*Arabidopsis* SR45 performs crucial roles in many areas of plant development and has also been implicated in responses to environmental cues [45,46,47]. To understand its function specifically in response to high-light stress, we first used PlantCARE to analyze the *SR45* promoter sequence (1100 bp immediately upstream of the ATG start codon) to identify the regulatory *cis*-elements that govern its expression. We discovered the *SR45* promoter to contain three light-responsive *cis*-elements along with three MYC-, two ABA-, one auxin-, and one MYB-responsive *cis*-element (Figure 1A). To further verify the function of these elements in light stress responses, we investigated the expression of *SR45* in RNA-seq data from [57], which examined transcriptome changes in seven-day-old *Arabidopsis* seedlings in response to high light (HL). We found that upon HL exposure, *SR45* expression is increased in the first 0.5 to 12 h, then declines from 12 to 72 h; moreover, while under moderate “growth light” (GL) there is no significant induction (Figure 1B). After hour 72, the HL-exposed seedlings were allowed to recover for 14 h in GL, during which SR45 returned to a baseline level (Figure 1B).

To verify this pattern of *SR45* expression under high-light stress, we performed qPCR analysis in mature plants (21 days old). The findings were consistent with the prior RNA-seq data, showing that *SR45* expression increased in response to HL (Figure 1C); however, this induction was not triggered until after two hours of light exposure, distinct from the seven-day-old seedlings. Together, these findings demonstrate that *SR45* expression is substantially controlled by light intensity, suggesting that SR45 may be crucial in light stress responses.

### 3.2. Under High Light, the sr45 Mutant Dramatically Accumulates Anthocyanin

**Figure 2:**
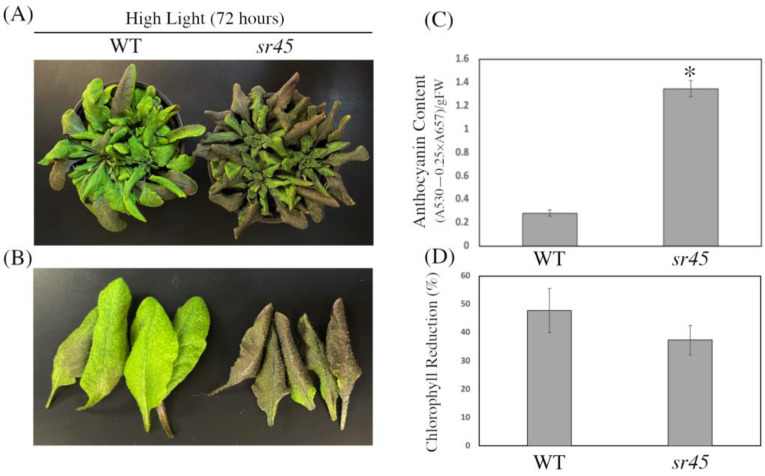
Response of the *sr45* mutant to HL. (**A**,**B**) Phenotypes of WT and *sr45* plants after 72 h of growth under HL (400 mol m^−2^ s^−1^ continuous light at 22 °C). Both complete plants (**A**) and close-ups of individual leaves (**B**) are shown. (**C**) Quantification of anthocyanins in the leaves of WT and *sr45* plants after 72 h of growth in HL. (**D**) Percent difference in chlorophyll reduction with HL treatment vs. GL. Asterisks indicate a significant difference (* *p* < 0.05) determined by a *t*-test.

In light of the above-described findings, we next set out to investigate how the *sr45* mutant reacts to intense photosynthesis-inhibiting light. We first cultivated WT and *sr45* plants in soil for 21 days under moderate light (“growth light”, GL), as specified in the Materials and Methods, and then exposed experimental groups to high light (HL) for 3 days (72 h) while maintaining control plants under GL. We found that *sr45* mutant plants accumulate significantly more anthocyanin pigments than WT plants after 72 h of light stress (Figure 2A–C). We additionally examined chlorophyll content under HL and found no significant difference between WT and the *sr45* mutant (Figure 2D). These findings indicate that loss of SR45 function enhances anthocyanin accumulation in response to high-light stress and suggests a negative function of SR45 in the anthocyanin biosynthesis pathway under HL.

### 3.3. Different SR45 Splicing Variants Respond Differently to High Light

**Figure 3:**
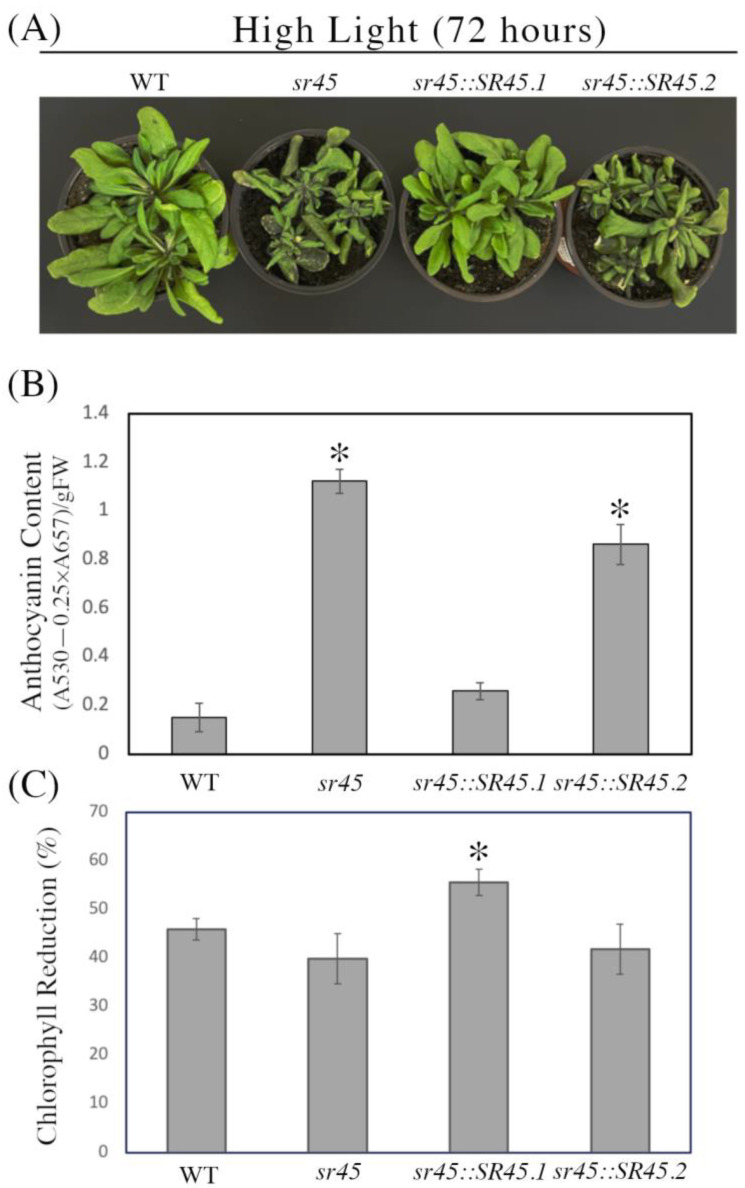
Responses of plants expressing individual SR45 isoforms to HL. (**A**) Phenotypes of WT, *sr45*, *sr45::SR45.1*, and *sr45::SR45.2* plants after 72 h of growth under HL (400 mol m^−2^ s^−1^ continuous light at 22 °C). (**B**) Quantification of anthocyanins in leaves from each genotype after 72 h of growth in HL. (**C**) Percent difference in chlorophyll reduction with HL treatment vs. GL. Asterisks indicate a significant difference (* *p* < 0.05) determined by a *t*-test.

It has been demonstrated that SR45 splicing variants have distinct functions in *Arabidopsis* development, and additionally, during stress responses [51,52]. Therefore, we aimed here to determine the specific roles of the two SR45 isoforms (SR45.1 and SR45.2) in light stress. Isoform-specific over-expression lines on the mutant background (*sr45::SR45.1* and *sr45::SR45.2*) were grown alongside WT and *sr45* mutant plants for 21 days under GL, and then experimental groups were shifted to continuous HL for 72 h while control plants were retained under GL. We found that the *SR45.2* over-expression line and SR45 mutant both exhibit significant anthocyanin accumulation in response to high-light stress as compared to WT and the *SR45.1* over-expression line (Figure 3A,B). However, when we evaluated chlorophyll levels, we found the *SR45.1* over-expression line to show significant chlorophyll loss under HL compared to all other lines (Figure 3C). These results indicate that the role of SR45 in HL stress responses is regulated by its AS even though the two isoforms are almost identical, differing by only a few amino acids. 

### 3.4. SR45 Binds to and Regulates Anthocyanin Biosynthesis Gene Transcripts

**Figure 4:**
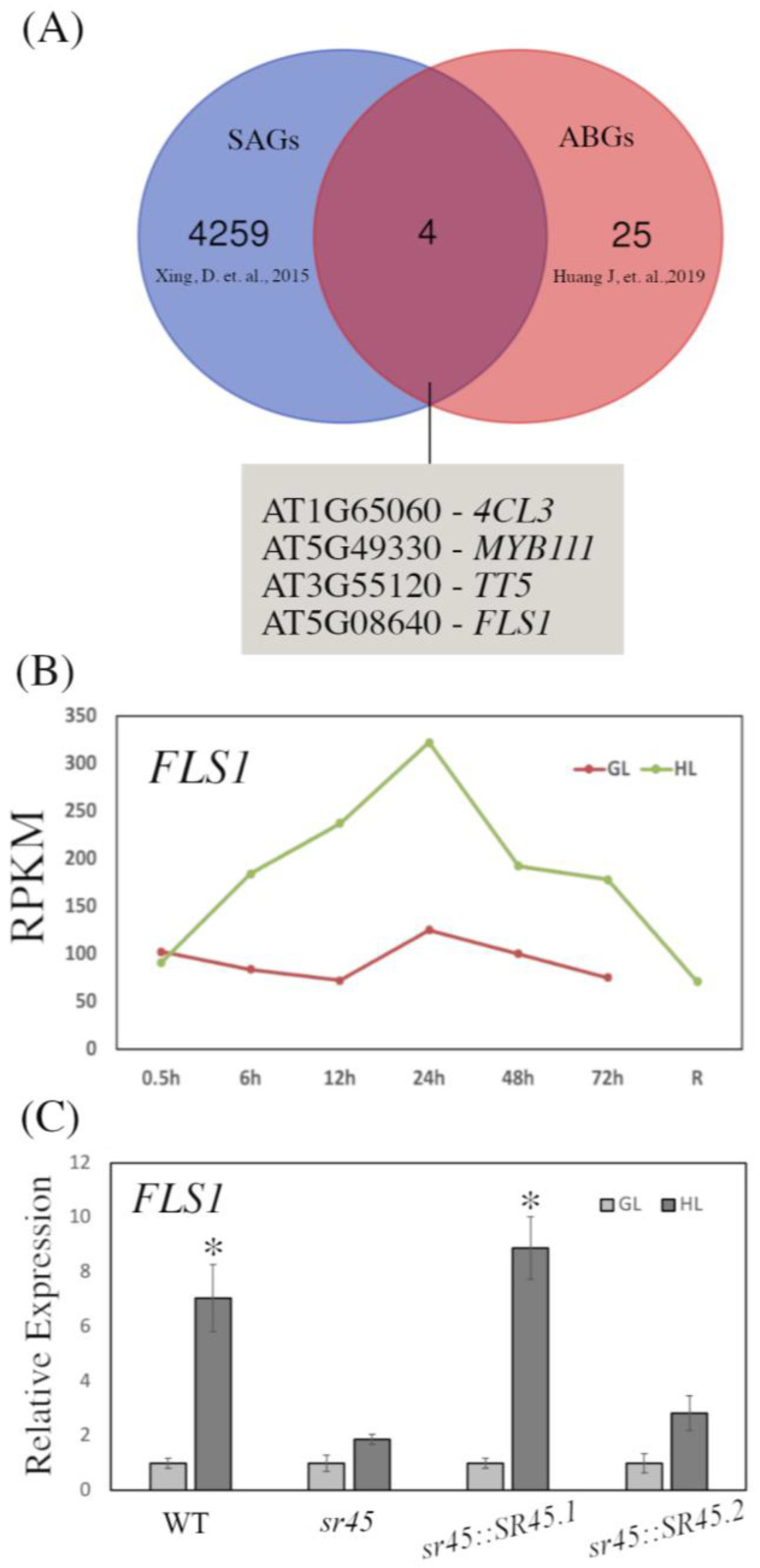
SR45 binds gene transcripts involved in anthocyanin production. (**A**) Overlap between SR45-associated genes [53] and anthocyanin biosynthesis genes [57]. (**B**) Expression of *FLS1* under HL and GL conditions, based on RNA-seq data [57]. The *x*-axis indicates the time points and after recovery (R). The *y*-axis represents the average reads per kilobase per million mapped reads (RPKM). (**C**) Relative expression of *FLS1* in all evaluated genotypes under HL, based on qPCR; the expression level under GL was set as 1. Asterisks indicate a significant difference (* *p* < 0.05) determined by a *t*-test.

A transcriptome-wide investigation of SR45 targets found it to bind to more than 4000 transcripts, the majority of which are genes involved in plant growth and stress tolerance [53]. The genes responsible for anthocyanin biosynthesis are known, with the majority having been thoroughly characterized [19,20,21,22,57]. Here, we intend to determine which genes involved in anthocyanin biosynthesis are likely to be regulated by SR45. First, we determined the overlap between SR45 targets (SR45-associated genes, SAGs) identified in [53]. We found that there are four ABGs also counted among the SAGs: *FLAVONOL SYNTHASE 1* (*FLS1*), *4-COUMARATE:COA LIGASE 3* (*4CL3*), *MYB111*, and *TRANSPARENT TESTA 5* (*TT5*) (Figure 4A). Since it has been demonstrated that the *Arabidopsis fls1* mutant shows increased anthocyanin biosynthesis [61,62], and the RNA-seq data indicate *FLS1* to be upregulated in response to HL (Figure 4B), we examined *FLS1* expression in WT, *sr45* mutant, and over-expression lines under GL as well as HL. We found *FLS1* expression to be significantly reduced in response to HL in the *sr45* and *sr45::SR45.2* plants as compared to WT and *sr45::SR45.1* (Figure 4C). These findings suggest that, in response to HL, SR45 binds to the *FLS1* transcript and regulates its expression at a post-transcriptional level, thereby adversely modulating anthocyanin production.

### 3.5. SR45 Mediates Anthocyanin Biosynthesis through the miR156-SPL Module

**Figure 5:**
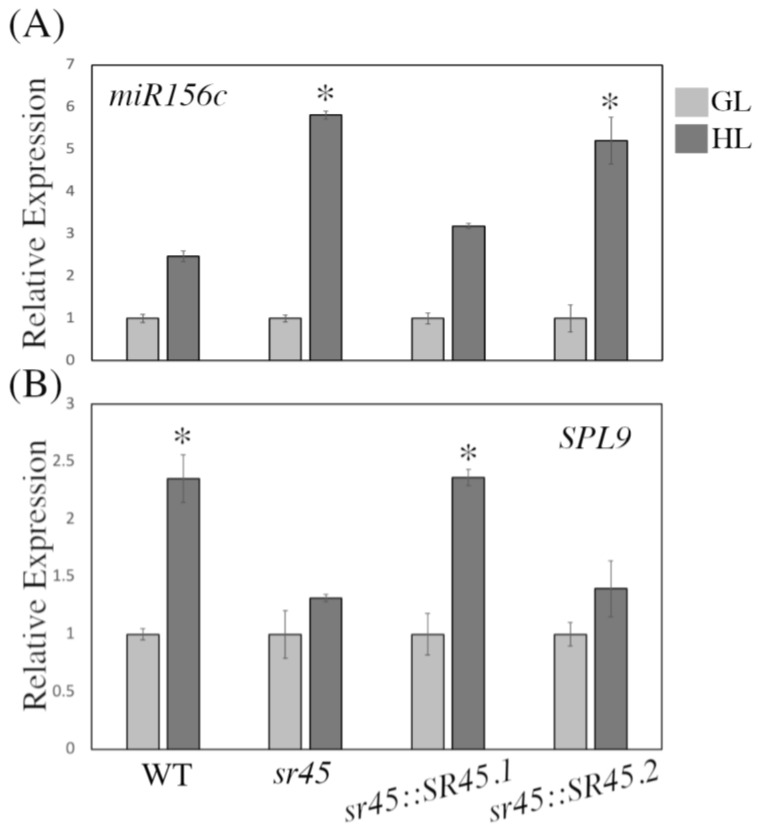
Expression of *miR156c* and its target *SPL9* in all evaluated genotypes. The relative expression of *pre-miR156c* (**A**) and *SPL9* (**B**) in all evaluated genotypes under HL was determined by qPCR; the expression level under GL was set at 1. Asterisks indicate a significant difference (* *p* < 0.05) determined by a *t*-test.

Anthocyanin biosynthesis is negatively regulated by the SPL transcription factor *SPL9*, which is a target of miR156 [29]. In addition, it is now understood that AS regulates the synthesis of most microRNAs in plants [63]. Accordingly, we aimed here to investigate the expression of miR156c and its target *SPL9* under GL and HL in all evaluated genotypes. We found that miR156c is upregulated in mutant and *sr45::SR45.2* plants under HL as compared to WT and *sr45::SR45.1* plants (Figure 5A), resulting in the down-regulation of *SPL9* (Figure 5B). These findings are consistent with the previous report that miR156 over-expression suppresses *SPL9* and indicate that SR45 also plays a critical role in regulating microRNA expression in response to HL which, in turn, impacts anthocyanin biosynthesis.

### 3.6. The sr45 Mutant Is Resistant to PQ-Induced Oxidative Stress

**Figure 6:**
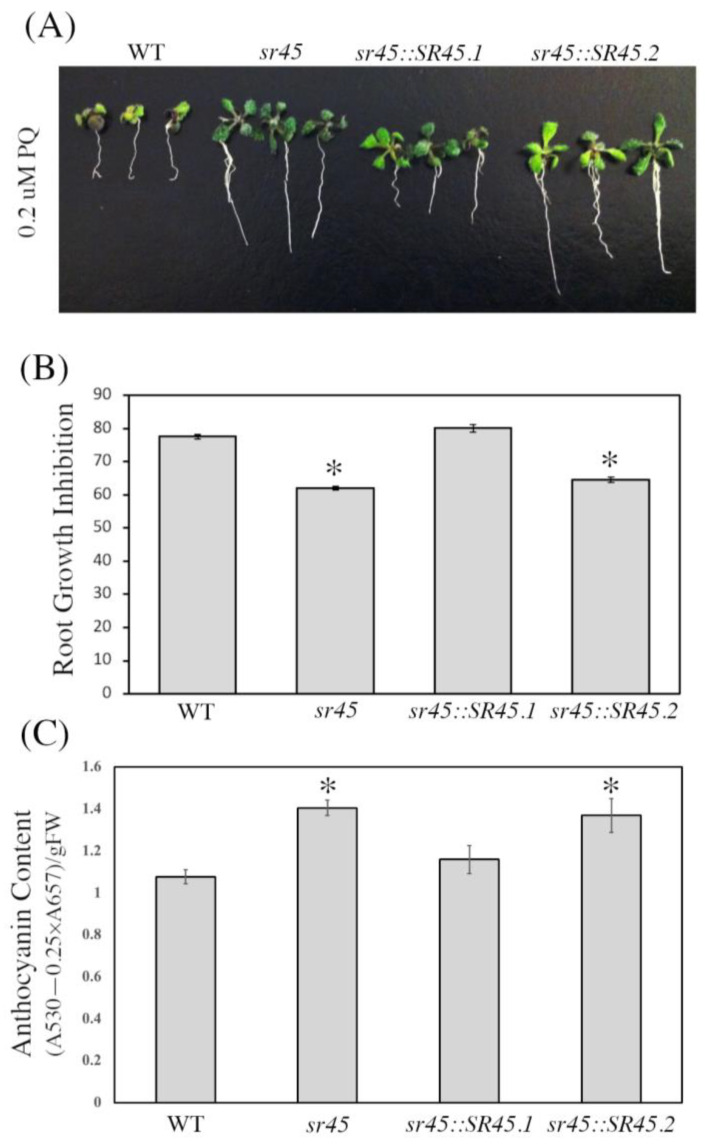
Responses of all evaluated genotypes to PQ. (**A**) Phenotypes of PQ-treated WT, *sr45*, *sr45::SR45.1*, and *sr45::SR45.2* seedlings. Seeds from all genotypes were germinated on Murashige and Skoog medium with or without 0.2 uM PQ for 2 weeks at a temperature of 22 °C with a light intensity of 120 mol m^−2^ s^−1^ and a light/dark cycle of 12:12. (**B**) Percent root growth inhibition in PQ-treated seedlings. (**C**) Quantification of anthocyanins in whole seedlings after two weeks of growth with PQ treatment. Asterisks indicate a significant difference (* *p* < 0.05) determined by a *t*-test.

Given that anthocyanin serves as an antioxidant and that its synthesis can be triggered by a variety of ROS-inducing substances, our next goal was to examine how the *sr45* mutant and individual isoform-complemented over-expression lines respond to treatment with the oxidative agent PQ. Following the procedures outlined in the Methods section, seeds of all genotypes were cultivated for 2 weeks on MS with or without 0.2 uM PQ, after which we examined root development in all lines. This revealed that the *sr45* mutant and *SR45.2* over-expression line have substantially higher PQ resistance as compared to WT plants and the *SR45.1* over-expression plants (Figure 6A,B). In addition, we found that under PQ treatment, *sr45* and *sr45::SR45.2* plants accumulate significantly higher levels of anthocyanin as compared to the other lines assayed (Figure 6C). Taken together, these results suggest that the *sr45* mutant and *sr45::SR45.2* transgenic line, which have higher levels of anthocyanin, may have a greater antioxidant response to ROS than WT plants or the *sr45::SR45.1* transgenic line.

## 4. Discussion

SR45 function has been demonstrated to be important to *Arabidopsis* growth and stress control. Here, we report that SR45 is a crucial component of how plants respond to HL. The accumulation of anthocyanins is a typical response that plants use as photoprotection, allowing them to adapt to high levels of illumination. Our results show that loss of SR45 function results in greater accumulation of anthocyanin in response to HL, while chlorophyll content did not notably vary between WT and *sr45* plants. We also found that SR45 binds to and controls the expression of gene transcripts involved in flavonoid production, such as *FLS1* and *miR156*. Characterization of PQ tolerance also suggested that *sr45* mutant plants are better able to endure increased ROS due to their high anthocyanin content, which acts as an antioxidant. Thus, we concluded that SR45 is a negative modulator of HL-induced anthocyanin biosynthesis and its functionality may affect the antioxidant capacity of plants under HL stress.

AS is known to promote plant adaptation to several environmental conditions, but the mechanism by which plants modify their RNA splicing in response to changes in light intensity is not well understood. The splicing factors RS31, SR30, SR34a, SR34b, and U2AF65a have all been implicated in the regulation of light-regulated AS in *Arabidopsis* [38,39]. Under HL stress, *Arabidopsis* SR45a has also been found to reduce splicing efficiency by creating a bridge between 5′ and 3′ splice sites [64,65]. Most of these splicing factors interact directly or indirectly with SR45 [52,65,66], which suggests that they may all work together to control anthocyanin biosynthesis under HL stress.

Importantly, two protein isoforms, SR45.1 and SR45.2, are produced from *SR45* through AS, and these isoforms may have either entirely different or related functions in plant development and stress responses. Here, we observed that plants only over-expressing *SR45.2* respond to HL stress similarly to the *sr45* mutant, but those over-expressing only *SR45.1* respond similar to WT plants. The two SR45 isoforms differ by just seven amino acids, the span of which contains two potential phosphorylation sites, suggesting that SR45 is regulated post-translationally in response to HL stress and that its function in anthocyanin biosynthesis may be mediated through protein phosphorylation.

It has been demonstrated that SR45 binds thousands of RNAs, indicating a broad role for this protein in post-transcriptional regulation of gene expression. In the present study, we discovered that SR45 binds to *4CL3*, *MYB111*, *TT5*, and *FLS1*, all of which are involved in the first steps of anthocyanin biosynthesis; this points to an early role for SR45 in regulating the process. Notably, *FLS1* is the only one of these targets for which mutation was associated with increased anthocyanin accumulation. As such, we examined *FLS1* expression in the *sr45* mutant which revealed a significant reduction under HL exposure. It merits mention that plants with mutations in *FLS1* or *SR45* share several abnormalities in development and stress responses, which underscores the function of SR45 in regulating *FLS1* expression. It is also important to note that the determinations of SR45 binding in this work were based on plants grown under normal conditions. Further investigation of the SR45 interactome in the HL context will allow for more accurate and complete identification of the SR45-regulated genes involved in the response to HL.

Micro-RNA synthesis is mediated by RNA splicing, with a number of miRNAs as well as their targets alternatively spliced [67,68]. Given that *miR156c* is alternatively spliced and known to regulate anthocyanin biosynthesis, we investigated whether it is a target of SR45. Our findings support miR156c as a mediator through which SR45 controls anthocyanin accumulation under HL. However, *miR156c* was not included in the SR45 interactome published in a prior work [53], which we believe was due to a technical limitation. It might be worthwhile to re-examine the SR45 interactome with an emphasis on miRNA transcripts, particularly under stress situations such as HL.

Finally, taking into consideration the fact that anthocyanins act as ROS scavengers and photoprotective agents to shield plants from the effects of oxidative stress, we examined the responses of SR45 mutant and over-expression lines to PQ, a ROS-inducing herbicide. We found that the mutant and the *SR45.2* over-expression line both accumulated high levels of anthocyanin in response to PQ, suggesting they are generally more resistant to PQ and oxidative stress. This conclusion was supported by root length measurements. Thus, our findings indicate that the *Arabidopsis* antioxidant system is severely impacted by HL due to the function of SR45 as a negative regulator of anthocyanin accumulation.

## 5. Conclusions

RNA splicing, a modulator of genome expression, allows plants to respond to light input by fine-tuning gene activity. Through interactions with spliceosomal proteins and target RNAs, splicing regulators such as SR45 play critical roles in controlling the light response. Here, we discovered that SR45 is involved in the response to high-light stress by controlling the accumulation of anthocyanin, an essential plant pigment that aids in photoprotection and antioxidant activity.

## Data Availability

Data are contained within the article or supplementary material. Additional inquiries can be directed to the author.

## Supplementary Materials

The following supporting information can be downloaded at: https://www.mdpi.com/article/10.3390/life13061386/s1, Table S1: RT-qPCR primers (Forward-Reverse, 5′ to 3′).
