# Supplementary material for: The Splicing Factor SR45 Negatively Regulates Anthocyanin Accumulation under High-Light Stress in Arabidopsis thaliana
Source: Life (Basel). 2023 Jun 14;13(6):1386. doi: 10.3390/life13061386 (PMC10303452; doi:10.3390/life13061386)
Supplement: Supplementary file 1 [file life-13-01386-s001.zip › life-2454269-supplementary.pdf]

Supplemental Table S1: RT-qPCR primers (Forward-Reverse, 5' to 3')

Gene ID	Gene Name	Forward Primer	Reverse Primer
AT1G16610	*SR45*	AAGTCCTGCTGGACCTGCTA	CCTTCTTCGAACAGGACTGC
AT5G08640	*FLS1*	ACACCATCCCCCAAATTACA	TTAGATCGACGACGGGAATC
AT4G31877	*miR156c*	AAAAGCCTCAGATCTAACTCCAACAC	GCGTTTCTCTTAAAATTTGTCCCAAAACT
AT2G42200	*SPL9*	TCAACAGTGCAGCAGGTTTC	TTCCATTCATTCCAGCATCA
AT3G18780	*ACT2*	GGCAAGTCATCACGATTGG	CAGCTTCCATTCCCACAAAC
